# Epigenetics Role in Spermatozoa Function: Implications in Health and Evolution—An Overview

**DOI:** 10.3390/life13020364

**Published:** 2023-01-29

**Authors:** Julia Andreu-Noguera, Andrea López-Botella, Paula Sáez-Espinosa, María José Gómez-Torres

**Affiliations:** Biotechnology Department, Science Faculty, University of Alicante, 03690 Alicante, Spain

**Keywords:** assisted reproductive techniques, epigenetics, male infertility, paternal inheritance, spermatozoa

## Abstract

The unique properties of spermatozoa are established through the spermatogenesis and maturation processes concurrently with its epigenome. It is known that damage to epigenetic mechanisms can lead to reproductive problems. However, scientific reviews addressing the role of the spermatozoa epigenome during the reproductive process are scarce. Therefore, the aim of this review was to offer a detailed overview of current knowledge in the field of spermatozoa epigenetics and its consequent implications. A full search was performed through three databases by combining five keywords. Inclusion criteria were implemented to grant accessibility, relevance, and concretion. Besides, some articles were manually removed or added to obtain an adequate and complete collection of 485 scientific publications. This compilation was used to conduct the bibliometric analysis and the data review separately. Bibliometric results displayed that spermatozoa epigenetics is an active and growing research area. The bibliographic overview showed that sperm epigenome correlates with the development of its function, explaining the environmental influence on reproductive pathologies or abnormal inheritance. The main conclusions were that the normal performance of sperm is heavily reliant on its epigenetics and that this study area is burgeoning, with the potential ability to provide society with clinical innovations in a short-term period.

## 1. Introduction

The first description of the spermatozoa appeared in the S.XVII by Antonie Van Leeuwenhoek, who described them as “spermatic animalcules”. Although environmental factors were extensively described in the S.XIX, it was only in the S.XX that the concept of “epigenetics” began to spread through many research areas in biological sciences. Thus, it became a bridge between external causal factors and their phenotypic consequences, which is particularly significant in the reproductive and development area [1,2,3,4].

**Figure 1:**
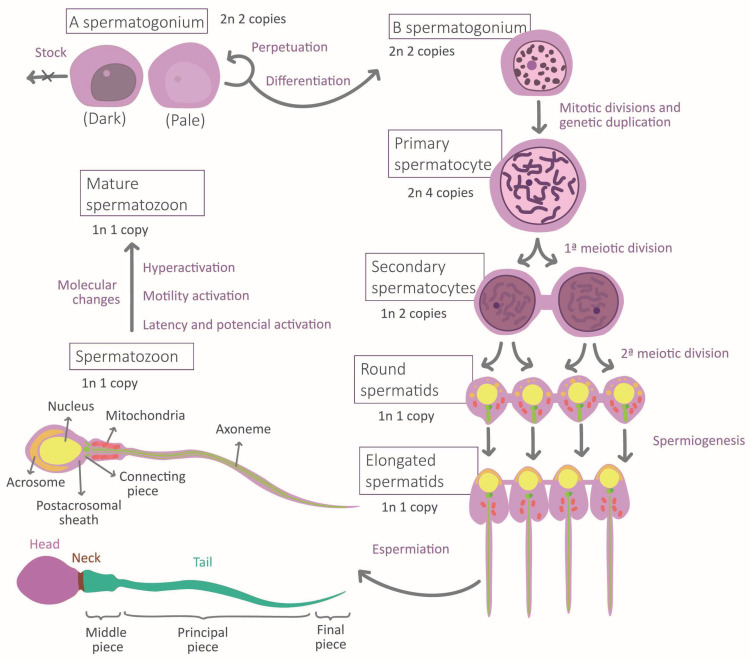
Stages of spermatogenesis and morphological changes associated with spermatozoa maturation.

During the migration of germ cells to the gonads in embryonic development, the cellular environment induces marked differentiation into gonocytes that will become spermatogonia after birth. Mature sperm requires normal spermatogenesis and subsequent maturation to acquire their fundamental morphological and physiological properties [5] (Figure 1). Spermatogenesis occurs in the seminiferous epithelium, in which bottom spermatogonia type A divide mitotically [6,7]. Spermatogonia destinated to differentiation transform into type B spermatogonia to originate all the successive transitory cell types [6], each one situated at a different level in the epithelium until the most apical level where spermatozoa are located [7]. The transformation consists of a meiotic division phase, which confers spermatozoa to its haploid nature, and a spermiogenesis phase [7]. This last stage is when the attribution of physiological and morphological characteristics to the sperm occurs: acrosome and intermediate piece conformation, flagella elongation, clearing of most of the cytoplasmatic content, and nucleus restructuration [7] (Figure 1). This process ends with spermiation, in which spermatozoa are released by the Sertoli cells (SCs) [7]. SCs function is to maintain and regulate the gametes along the epithelium, to limit population growth, and to select the most fitness cells via apoptosis [5,8]. Another important type of cell is Leydig cells, placed between the seminiferous tubules, which regulate spermatogenesis by testosterone liberation [7].

The maturation and fertilization capacity develops along the journey of the spermatozoa from the seminiferous epithelium to the encounter with the oocyte. It leads the male gamete to a state of hyperactivation, which guarantees that the action of the spermatozoa occurs at the moment adequate and with a sufficient intensity of motility [9]. The most remarkable changes occur during epididymal storage, where a molecular interchange is established between the gametes and the epididymal epithelium, which results in spermatozoa and plasma seminal molecular content transformation [10]. A significant interchange with the reproductive feminine apparatus epithelium also takes place [10], which prepares the sperm to find and interact with the feminine gamete [11,12]. In this stage, the extreme physicochemical and biological conditions will again force the selection of spermatozoa by competition [13,14]. Oocyte recognition would induce the last activation boosting, causing the final selective stage in which one spermatozoon would penetrate the pellucid zone and transfer its content to the feminine gamete [15].

The spermatozoa morphological and physiological high level of differentiation respond to the high selective pressure to which it is subjected while developing its function [14]. Its properties are aimed at storing and protecting its nuclear and remaining cytoplasmatic content while its information is transferred to the oocyte [8,16,17]. Specifically, the high compaction of the DNA protects genetic material from degradation [8,18]. Together with the scarce presence of cytoplasm, they result in a head with a minimal volume and a highly hydrodynamic shape [8,17,18]. This facilitates its displacement, which is only possible due to the energy proportionated by mitochondria [13] and to the flagella structure [5,7]. Spermatozoa interaction with the medium would lead this movement to follow the oocyte by chemotaxis until gamete recognition [12]. That contact triggers the acrosomal reaction, which degrades the pellucid zone and allows spermatozoon to deliver its content to the oocyte [15]. Part of this content, the post-acrosomal sheath molecules, will activate oocytes [19]. Overall, the individual roles of each part converge on the spermatozoa principal function: fecundation. Two additional functions derive from it: embryonic development regulation and inheritance [8]. 

The correct establishment of sperm properties is subject to the acquisition of suitable epigenetic patterns [17]. The concept of “Epigenetics” has had many different definitions [1,4,20,21]. A possible definition of epigenetics is that it is the set of phenomena by which a group of mechanisms (epigenome) regulates expression without altering DNA sequence [22] by changing the accessibility or availability of elements involved in the transcription and translation processes [17]. These mechanisms respond to environmental conditions, either the extracellular or the external environment [10,17,23,24,25]. Expression patterns define each cell type and its intratypic variation, that is to say, the phenotypic identity of the cell [1,26]. Taken together, epigenetics represents an additional information layer to the genotype and is dependent on the environment [26,27]. The epigenetic activity derives from transcriptome and proteome [3,28], and the traditional epigenetic mechanisms (chemic DNA modification and chromatin package [16]) are a result of thereof [3,28]. Currently, these traditional mechanisms are studied independently, and transcriptome and proteome research with an epigenetic orientation is a minority. 

For pragmatism reasons, this review has considered four individual mechanisms (the methylome, the packaging proteins, the transcriptome, and the proteome), even though their activities are tightly related and overlapping. On the one hand, DNA methylation patterns and the histone variants distributions and modifications are associated with the variation of accessibility to chromatin regions and, consequently, with the differential expression level [3,5,6,8,10,17,18,28,29,30,31,32]. On the other hand, the transcriptome and the proteome intervene at many regulatory levels by interacting with chromatin and between them [3,5,6,8,10,17,18,28,29,30,31,32]. For further information on epigenetic mechanisms, consult Table S1 and Figure S1 in the Supplementary Materials. The interdependence of those epigenetic mechanisms allows information interchange, which is beneficial in unstable situations or when one of them has its integrity compromised [17,31]. Besides such dependence, effector agents (RNAs and proteins) are also subordinated to the DNA sequence. Therefore, genetic alterations can result in epigenetic disrupted activity [25,33]. In the same way, the expression of epigenetic patterns influences DNA sequence, as it happens when the mutation probability increases due to the presence of methylation marks or when DNA fragmentation occurs caused by an erratic chromatin package [4,21,25,33]. 

Some studies signalize that the performance and coordination of epigenetic mechanisms in spermatozoa have implications for health and evolution [4,10]. Specifically, reproductive problems are a concerning and growing health problem affecting around 15% of couples worldwide, where male partners are responsible for 20–30% of the overall infertility cases [34]. The decline of male fertility is a worldwide matter of concern since available studies suggest a lower semen quality over the years [35], thus being related to infertility problems. To deal with these situations, assisted reproduction techniques (ART) have been developed to help to solve part of these problems.

The value of the spermatozoa epigenetics study lies, beyond the academic contribution to Life Sciences, in its clinical applicability through many aspects of the field of reproductive and developmental biology: from the improvement of gametes selection and the preparation techniques to the organism intra and extrauterine development monitoring, including diagnosis and treatment of diseases, among other examples [8,21,36,37]. Considering the importance of sperm epigenetics during the reproduction process, the main objective of this review was to compile updated global information on this field of research from works published between 2011 and 2022 to offer a panoramic of the accumulated knowledge and the state of the research. 

## 2. Materials and Methods

With the aim of selecting papers that fit the assessed topic and finding suitable amounts for the bibliometric analysis and the brief review, a search protocol was established. First, an approaching search was carried out through several databases using diverse keywords. Based on that, three databases were chosen (Scopus, PubMed, and Web of Science), and five keywords were selected and optimized: “Epigenetics”, “Spermatozoa”, “Male infertility”, “Paternal inheritance”, and “Assisted reproductive techniques”. 

The number of articles that responded to each keyword combination quest was annotated in a document, and the different combinations were compared. Thereupon, six useful combinations were chosen to limit the number of search results. Consecutively, definitive searches were accomplished by alternating several match modes in the databases using six combinations of the keywords. The number of articles obtained was again registered. Due to the quantity and quality of results, it was decided to only use the search that employed the match mode TAK (Title, Abstract, or Keywords) for further data recompilation.

**Figure 2:**
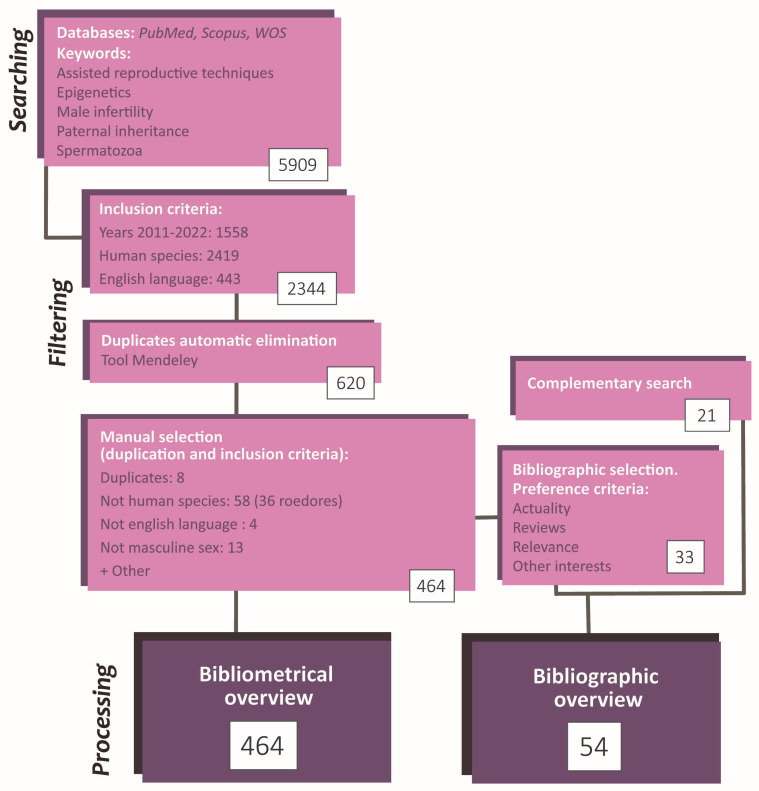
Flowchart summarizing the search and selection process of the articles included in the present article. The study methodology comprises a searching phase, a selection and record phase, and a phase of statistic data analysis and conceptualization. The total number of articles employed was 485 (464 by initial search + 21 by complementary search).

These papers were filtered based on inclusion criteria (IC). IC was: (i) reading accessibility (written in English), (ii) published between 2011–2022, (iii) performed in humans. The number of results applying each individual IC was simultaneously recorded. Then, the bibliometric parameters of the selected articles were exported to Mendeley, and software tools were used to discard duplicated papers. The remaining duplicates were manually eliminated. In parallel, articles that did not fit the topic orientation (not in masculine sex or not about the reproductive area) or the IC mentioned above were also manually discarded. For each article, additional parameters were fulfilled: type of study and authors; country and institution in which it was carried out; journal and editorial that published it; and citation rate. Further to this, their study orientation and epigenetic mechanisms were checked and annotated. The analysis consisted of simple computations and the elaboration of tables and graphics reflecting relevant information. The most significant figures were selected to be included in this study (Figure 2).

Finally, for the review purpose and from the selected papers, some articles were carefully chosen based on actuality, number of citations, and content. Besides, some searches were performed to complete specific information in the article compilation. It is worthwhile to notice that these additional articles do not need to fit the inclusion criteria since they were not used in the bibliometric analysis. Consequently, the information in the review does not refer exclusively to the human species (Figure 2).

## 3. Results

### 3.1. Bibliometric Results

The search strategy and inclusion criteria employed, as well as the posterior analysis, allowed for obtaining interesting bibliometric results and inferring some tendencies in the study of the human spermatozoa epigenetics. Beyond the selected and exposed figures, the Supplementary Material can be consulted an additional figure (Figure S2) and tables (Tables S2–S5).

**Figure 3:**
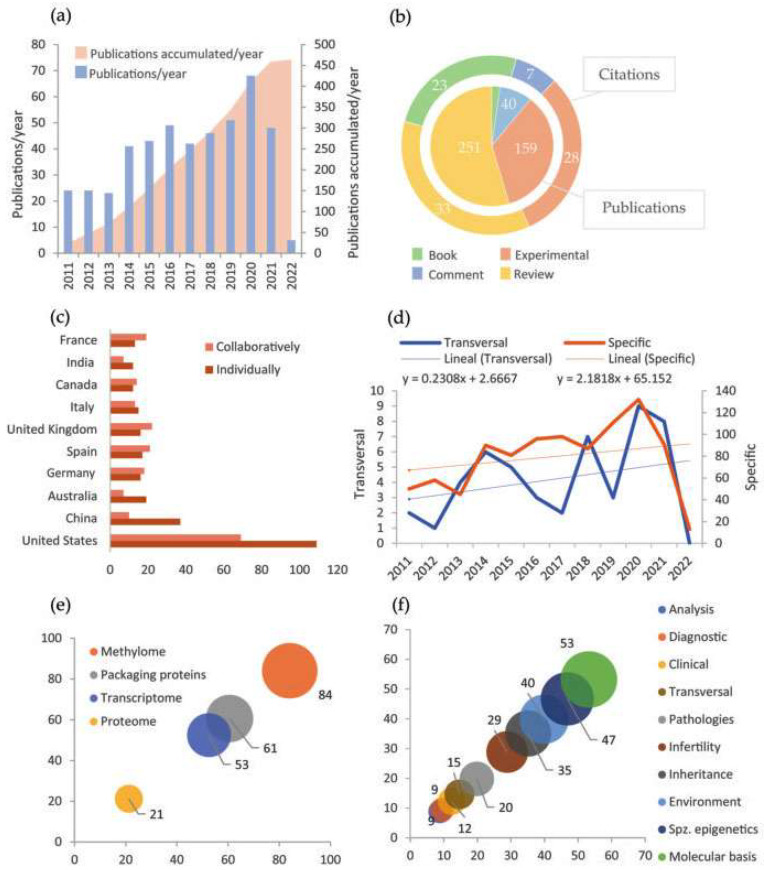
Main results of bibliometric analysis. (**a**) Variation of the number of annual publications (blue) and accumulation of publications (pink) in the period 2011–2022; (**b**) Number of publications and citations to each type of document; (**c**) Number of publications of each country carried out individually (brown) or in collaboration with other countries (pink). There only appear the countries with more than ten studies carried out individually; (**d**) Variation of the number of annual publications with specific orientation to epigenetics (brown) and with a transversal study of epigenetics (blue). The dashed line and the equation show the linear tendency. Notice that they are expressed at a different scale; (**e**) Percentage of publications that address each of the mechanisms in spermatozoa centrally; (**f**) Percentage of publications oriented to study each of the aspects mentioned about the spermatozoa epigenetics (spz. = spermatozoa).

Scopus was the database that greater results generated using the proposed IC and keyword combinations (Table S2, Supplementary Material). Further to his, the most frequent keyword used was “Epigenetics”. Its combination with “Male infertility” was the most commonly found in the papers, in contrast with its match with “Paternal inheritance” (Table S3, Supplementary Material). That gives us an idea of the study areas in which Epigenetics currently has a strong presence. The manual revision proved the database filters efficacy since very few had been wrongly admitted (Table S4, Supplementary Material). Besides, the number of papers discarded by each exclusion criterion indicated that this area of study tends to be realized in animal models, probably through basic investigation or preclinic phases; and most of them were written in English in the last decade (Table S4, Supplementary Material). Specifically, Figure 3a shows an increase in the number of annual publications during the period. All the appreciations are coherent with the normalization of assisted reproduction and the epigenetics transversal expansion to other areas.

Among all documents obtained, bibliographic reviews stand out, as displayed in Figure 3b. That points out the existence of huge, accumulated knowledge and a great effort centered on consistently organizing experimental information. Moreover, cites average by article indicates that book chapters are also a frequent source of documentation. Furthermore, Figure 3c evinces a dominance of the United States and China in publication numbers, either in single or collaborative studies. European countries, which also have a considerable presence, tend to develop international collaborative projects (Figure 3c). Although not shown in the graphic, the remarkable institutions concerning the number of publications are Utah University (United States) and Barcelona University (Spain). Some additional data to mention is that Jenkins, T.G.G. is the most active author in publication number, while Carrell, D.T. is the most cited on average (Table S5, Supplementary Material). Similarly, the journals that more often hosted a greater number of papers were respectively “Fertility and Sterility” and “Andrology”, as well as the publishers “Springer Nature” and “Elsevier”, all of which have high international relevance (Figure S2, Supplementary Material).

On the other side, section Figure 3e exposes that the more studied epigenetic mechanism among the four considered is DNA methylation, quadruplicating proteome study with epigenetic bias. This probably responds to historical reasons since methylation was the first mechanism whose epigenetic properties were described. Moreover, in Figure 3f, it can be appreciated that studies seem to be oriented to epigenetic molecular basis and spermatozoa epigenetic particularities. In contrast, applied studies (analysis techniques, diagnostic, treatment, and prevention) are rare. The appreciations in Figure 3e,f are consistent with a study realm at an early stage, which still requires establishing solid principles. Finally, Figure 3d shows that the evolution of the number of publications during the period 2011–2022 is different between the papers focused specifically on spermatozoa epigenetics and those whose approaches are transversal to other areas, being more accelerated the production of the first ones.

### 3.2. Review Results

The effect of the environment on sperm genesis and maturation through its epigenetics influences the reproductive health of men and the heritage of their offspring [27,38,39]. The following sections expose the spermatozoa normal epigenetic particularities, how it is established, and its role in spermatozoa functions. Additionally, it is addressed how the environment can modify epigenetics and the evolutive implications involved.

#### 3.2.1. Spermatozoa Epigenetics

##### DNA Methylation

Regarding DNA methylation, even though the human spermatozoa methylome is heterogeneous between samples or even between a subpopulation of cells [40], it possesses certain high specific patterns with minimal variability [29]. Most of the variable marks locate in promotors and repetitive elements [16]. Spermatozoa are widely methylated, although some regions, such as promotors, are poorly methylated [16]. Likewise, the standard marks in CpG methylation have also been found in other dinucleotides in sperm cells, frequently in transposons [33]. Another singularity is that its imprint is homogeneous since the female imprinting marks were removed during its genesis [16]. The importance of this mechanism is evidenced by the elevated regulation level the marks are subject to, being conserved even in methyl group scarcity thanks to the overexpression of some enzymes [16,22].

##### Packaging Proteins

**Figure 4:**
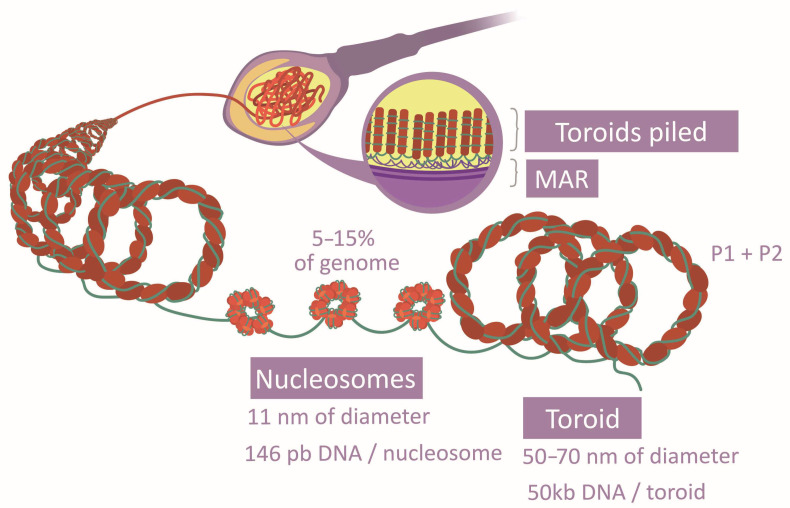
Comparison between histone and protamine compaction.

The spermatozoa DNA compaction relies on a unique system based on protamines, which are light proteins with a higher alkaline nature—greater than histones—due to the abundance of Arg [16,33,41]. This property allows them to drastically neutralize DNA acidity and generate a specific structure with a higher level of compaction (toroids) [33,41,42] (Figure 4). They grant the head an elevated density and a reduced size [22]. Two types of protamines exist (P1 and P2), whose proportion and location follow concrete patterns along the genome [33,42]. P1 forms loops of 11 pb, while P2 loops are formed of 15 pb. Further to that, their position in the DNA arches generates a marked curvature. The aggregation loops shape the toroids of 50 kb, separated and distributed by matrix attachment regions (MARs) [28,33,41]. Post-translational modifications of protamines also vary their affinity to DNA [29]. The free Cys and His or the zinc fingers establish disulfide bridges inter and intraprotamines, increasing the complex stability [33]. Although this is the most common chromatin structure in spermatozoa, histones there have also been described in certain regions [16,30] (Figure 4), and histone-modifying enzymes have been observed in spermatozoa [18]. Specifically, several histone variants and modifications are characteristic of this cell type [16,28]. It has been verified that there are histone variants, distribution patterns, and post-translational modifications associated with normal sperm cells [29]. All these aspects suggest that histone conservation is functional rather than random [16,30,33]. Surprisingly, histones have also been observed in the intermediate piece. However, their function is not clear as they may derive from previous stages or from mitochondria themselves, or they could have no function [18].

##### Transcriptome

Although until recently, it was considered that sperm transcriptome was a remanent from previous periods [17]. It is now recognized as an essential epigenetic mechanism [17,28]. Even if there is no agreement in relation to spermatic transcriptome composition in comparison to somatic cells, it is known that their proportions differ [17,28]. Spermatozoa possess a complex and characteristic group of RNAs, some of them exclusive of this cellular type [17,18,28]. Location and proportion patterns exist and are associated with normal spermatozoa, even though it presents some variability [28]. Small non-coding RNAs (sncRNAs) have a remarkable role, but there is no consensus about the relative abundance of their subtypes. A variety of mRNAs and long non-coding RNAs (lncRNAs) appear, too [8,22,28,29]. Furthermore, some RNAs are complete and functional, while others are fragmented [28]. Moreover, they can be protected by vesicles in ribonucleoproteins or remain free [10,28]. They can also increase their stability by adopting three-dimensional structures [10,17,28,30]. Regarding location, RNA locates in the head or tail and in the seminal plasma [18,30]. In the sperm head, RNA distribution is found between the cellular compartments and in the extranuclear and intranuclear membranes and can interact with chromatin [17,18]. In the tail, in addition to the RNA shared with the head, mitochondrial RNA also appears [18]. The extracellular RNAs could be considered a part of the transcriptome as they intervene in spermatozoa function [10]. 

##### Proteome

Focusing on the spermatozoa proteome consists of a diverse group of proteins with both cis or trans activity types. It should be noticed that at least 50% of them possess potential epigenetic activity (chromatin organization, histone modification, RNA interaction, etc.) [17]. While the transversal role of the proteins mentioned in the previous epigenetic mechanisms is widely known [3], their independent activities have scarcely been studied.

#### 3.2.2. Epigenetics during Spermatozoa Genesis

**Figure 5:**
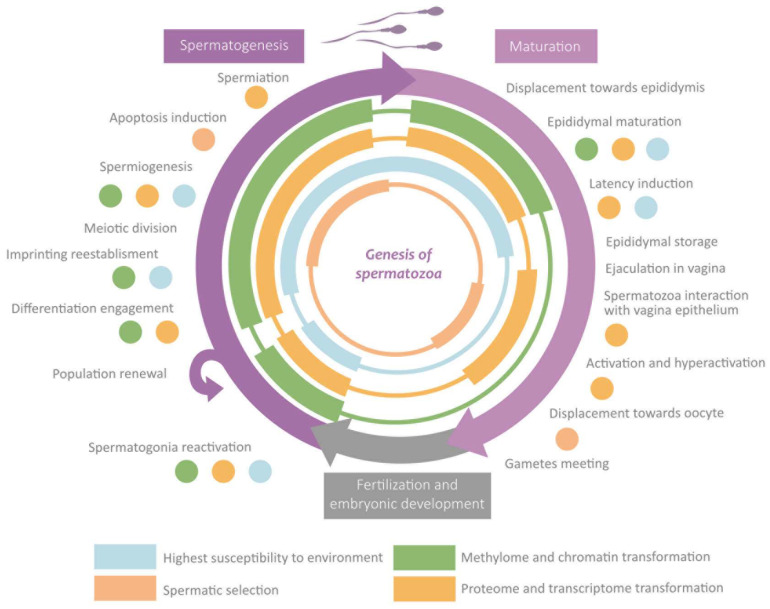
Overview of the role of epigenetics in the main events during spermatogenesis and maturation.

Spermatozoa epigenetic marks are established along the different stages the cell undergoes, which set its morphology and physiology [6]. Such modifications are induced by the extracellular environment, which can be influenced by the organism’s external environment, especially during the susceptibility windows [16,17,27]. The transmission through generations of epigenetic marks is limited by two epigenetic reprogramming events [33]. The first of them is germinal line reprogramming, which allows for establishing homogeneous imprinting. The second one is the preimplantation reprogramming which confers totipotencial properties to the zygote [1,16,27,33]. Although “reprogramming” usually refers to the waves of demethylating and remethylating, during this process also, significant changes take place in the rest of the mechanisms (histones-protamines transition, wide RNA loss, etc.) [17,27,28,33]. In the following sections are exposed the main changes that take place in each phase along with the susceptibility windows and the reprogramming events. These sections are summarised in Figure 5.

#### 3.2.3. Spermatogenesis

##### Germ Cells during Embryonic Development

During and after germ cell migration, the reprogramming events take place in the gamete, including active demethylation [16,33]. The marks are established again through the following stages by de novo methylation [33]. Consequently, 70% of the methylation marks of the embryo will not be transferred to the next generations [33]. This period is considered the first susceptibility window to environmental factors due to the enormous complexity that it involves [27,33].

##### Initiation of Spermatogenesis

Accelerated remethylating during preadolescence will reactivate spermatogonia, entailing a second susceptibility window [16,17,27]. Dialogue between RNAs, methylome, and histones would determine the expression patterns which lead to the transition across several cellular stages and the differentiation compromise [5,18,39]. For example, undifferentiated spermatogonia lack H3K9m2 (double methylation in Lys 9 of histone 3) and possess H3K9m3 (triple methylation in Lys 9 of histone 3) in the periphery. At the same time, both modifications are expanded for all the nuclei during differentiation [17,18]. This modulating dialogue is taken after by SCs cells [5], whose apoptotic effect depends on certain methylation marks during spermatozoa development [18]. Besides, maintaining the intercellular bridges during the process will allow a synchronic transformation [7], maybe including the epigenetic content. 

##### Chromatin Reorganization

Chromatin compaction is reached by the establishment of the toroidal structure based on protamines [18] and the incorporation of spermatozoa-specific histone variants instead of the remaining testicular variants [8,17]. The transition toward toroidal conformation begins with testicle histone modifications, being mostly replaced by transition proteins (TP1 and TP2) [18,22,33]. Some examples of modification are the H4 (histone 4) hyperacetylation, which makes chromatin aperture possible, or the addition of butyrate groups, which prevents histone substitution [17,22,29,33]. Finally, transition proteins are interchanged by P1 and P2 in a 1:1 ratio, also suffering tail modifications [18,22,29]. In such a way, the spermatic nucleus has an alternation between nucleosomes and toroids [17]. Histone-conserved regions, 5–15% of the chromatin (Figure 4), would maintain a flexible and transcriptionally accessible character [16,18,22]. Probably, the differential tendency of modification type of each histone variant explains why some of them are more frequently conserved [16]. There is evidence that transcriptome and proteome intervene in this transition and the ulterior maintenance of chromatin structure [22,28]. Besides this chromatin remodeling, a gradual transformation of the packaging proteins modification patterns takes place during spermatozoa genesis [18,33].

##### Methylome Reestablishment

During spermatogenesis, 4% of cytosines and 70% of CpG islands will be methylated, especially in regions with remaining histones or with repetitive elements [33]. In fact, histone modifications may have a relevant role in this process [16]. Due to the severity of this change, this is considered the third susceptibility window to environmental factors [27]. The set of masculine imprinting patterns established will drive hypermethylated spermatozoa compared with the female gamete, though both still are hypomethylated compared to somatic cells [16,17,33,43].

##### Proteome and Transcriptome Transformation

At the beginning of the spermatogenesis process, piRNAs (Piwi-RNAs) are the most abundant RNAs, followed by miRNAs (microRNAs) and tsRNAs (tRNA-derived small RNAs) [10]. However, at the end of the process, tsRNAs are at the maximum concentration while piRNAs are reduced [10]. These fluctuations in transcriptome composition, as well as the undergone by proteome, reflect its role in meiosis and morphogenesis regulation, along with the preparation for posterior phases [6,18,44]. For example, it has been observed that genes involved in pluripotential maintenance, in spermatozoa structure or metabolism, are under miRNAs or piRNAs control [6,18]. A remarkable fact is that, from the round spermatid phase with a compacted nucleus, the cell would be transcriptionally silenced, depending on preformatted transcripts [28]. Similarly, the cytoplasm reduction entails the loss of a great part of its proteome and transcriptome, also becoming translationally inactive and depending on the resynthesized proteome [33]. This is not a totally silenced situation since mitochondria seem to maintain certain expression activity [18]. 

#### 3.2.4. Maturation

##### Epidydimal Transit

A significant part of the molecules involved in the interchange between spermatozoa and epididymal epithelia are epigenetic elements [10]. Globally, the transcriptome increases and undergoes dramatic transformations [10]. For that reason, in the head and in the tail of the epididymis, the spermatozoa transcriptome shows different compositions [8,9]. The most prominent changes are the reduction of piRNAs, the increase of the tsRNAs, and the increase and diversification of the miRNAs [10]. Proteome, in turn, seems to have an important influence on the induction of latency and potential capabilities [9]. Some RNAs and proteins remain in the seminal fluid performing their roles [30]. Further to this, it has been noticed that in this period, methylome changes and higher stabilizing interactions in chromatin take place, probably derived from the proteome and transcriptome permutation [9,29,32]. 

##### Transit through the Female Reproductive System

The interchange of molecules between spermatozoa and other cells after ejaculation usually are not taken into consideration. However, the epigenetic modulation of spermatozoa also occurs by the maternal reproductive system lining epithelium, by its vaginal microbiota, or even by biological material deposited by previous sexual partners of the woman [10]. Besides, has been hypothesized about the existence of a collaborative interchange of epigenetic information between spermatozoa [10,33]. It is possible that during this transit, some methylation patterns change since methylation modifiers enzymes have been found in mature ejaculated spermatozoa [33]. Finally, the physicochemical conditions also modify spermatozoa epigenetics. For example, the influence of zinc concentration on protamines takes part in the activation and hyperactivation process [12,33].

#### 3.2.5. Epigenetics in Spermatozoa Functions

**Figure 6:**
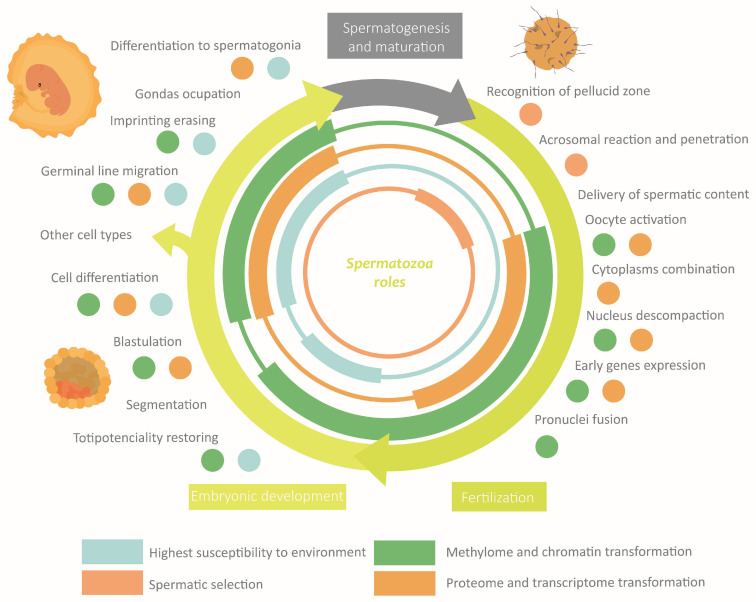
Overview of the role of epigenetics in the main functions of spermatozoa.

The correct establishment of the epigenome confers to spermatozoa the properties required to perform its specific functions [8,29]. Fluctuations in the external or the corporal environments can disrupt, through the extracellular environment, the spermatic epigenome [8,10]. An anomalous performance of spermatozoa leads to defects in fertility, embryonic development, or descendant phenotype [29]. Actually, it is hypothesized that a great part of idiopathic infertility cases would have an epigenetic origin, being more frequent than the exclusive genetics ones [22,29,45]. It is worth highlighting that the shorter latency period of the spermatozoa compared with the oocyte makes the probability of gaining epigenetic modifications smaller [23,46]. The main roles of epigenetics in sperm functions and how they can be altered are discussed below. These sections are summarised in Figure 6.

##### Fertilization

To achieve oocyte fecundation, spermatozoa must protect their genetic content and meet with the female gamete [8,17,18]. Then, the sperm cell has to bind to zona pellucida and fuse with oolemma [15]. For these purposes, it is essential a normal sperm morphology, chromatin compaction, activation and hyperactivation capacity, and the necessary sperm binding proteins [7,15].

Regarding methylome, its qualitative and quantitative variability in the sperm samples is quite higher in patients with fertility problems than in normozoospermic people [29]. Abnormal methylation has been associated with diverse altered seminal parameters (population density, maturation, morphology, motility, fragmentation, etc.) and with cases of oligozoospermia, teratozoospermia, azoospermia, asthenozoospermia, etc. [16,17,22,40]. Furthermore, aberrant methylation, including imprinting changes [17,22,43], seems to be one of the most common characteristics in sterility cases (41% of the cases undergoing ART) [29]. These disruptions are mainly triggered by the alteration of the enzymes that administrate the methyl groups [8]. Regarding chromatin structure in the spermatozoa nucleus, its impartment influences either the individual fertility or its descendants [8,22,32]. Quantitative or qualitative anomalies of protamines and histones distribution and modification patterns are common causes of low seminal quality [5,17,18,29]. They usually are a consequence of the temporal or spatial discoordination of the histone-protamine transition [18]. A recurring result is the disbalance of the P1:P2 proportion [16,22,29], leading to defective compaction, abnormal morphology, and DNA fragmentation [17,22]. Although appearing less frequently, there has also been identified differences between the transcriptome of normozoospermic and infertile men [8,17]. The perturbation of its proportions or locations, especially of miRNA, siRNA (small interfering RNA), piRNA, tsRNA, and rsRNA (rRNA-derived small RNAs), conduct to low spermatic density, immaturity and abnormal morphology and motility [6,8,9,17,28,31]. These defects can appear during the spermatogenesis or the epididymal stage and are prompted by either the genomic sequence mutation or due to complementary or regulatory proteins mutation [10]. Finally, protein ubiquity makes proteome involved in almost all reproductive problems, either in a complementary way to the previously mentioned mechanisms or with a central role [9,10,17]. 

##### Embryonic Development

The zygote changes that induce division and differentiation depart from a common point: the oocyte reactivation by the spermatozoa. The molecules transferred to the oocyte cytoplasm during fecundation include epigenetic elements [10,16,17,44]. Transcriptome and proteome, acting in trans faster than cis-elements, will trigger the initial response: meiotic reactivation and oocyte blocking to avoid polyspermy [8,16,47]. For instance, many spermatozoa RNAs code for or interact with development proteins [8,10,28,31]. One example is miR-34c, the most abundant miRNA in sperm, which is needed for the first embryonic division [33]. Furthermore, paternal chromatin undergoes a new reorganization to form the masculine pronucleus: protamines substitution by female histones reducing the compaction level, new histone modifications, etc. In brief, all above results in increased chromatin accessibility [18]. Simultaneously, the maintained histones allow direct access to their occupied regions by the expression machinery, contributing to early gene expression [16,17,27]. This is possible thanks to its lower compaction and its association with low methylation zones [16]. Some studies point out that regions with histones contain imprinted genes, ncRNAs, or homeobox genes, while others indicate that they are regions with low gene density (intergenic, repetitive, or retrotransposon) [16,18,22,27]. During development, the three-dimensional structure of chromatin and its distribution in the nucleus will change in each cell, defining each cell type [48]. The beginning of the development would require the reset of methylation marks by early active and late passive demethylation [16]. This remodeling is considered the fourth and last susceptibility window [27]. The marks that avoid the erasing will be present in the new somatic cells together with the newly acquired marks. Among the conserved marks figure the imprinting marks [16]. Its relevance resides in the functional inequivalence of the maternal and paternal epigenomes, which makes both needed for the proper development of embryo and placenta [1,18,37,43,49]. There seems to be a tendency to induce growing by the paternal epigenome, while the maternal one maintains it under control, generating a balance [1,43]. To sum up, the spermatozoa epigenome, cooperating with the oocyte epigenome and the newly acquired marks in the embryo, will orchestrate the tissue and organ differentiation. 

Perturbations in the spermatozoa genesis, even if they do not affect fertility, can still influence the embryo through epigenetics [9,28,50]. It has been demonstrated that disturbances in methylome, packaging proteins, transcriptome, or proteome conduct abnormal development and pregnancy loss [8,9,16,17,23,28,43]. Some of the most common origins are imprinting mismatches and the P1:P2 disbalance [16,17,28]. It has also been proved that the restoration of the normal epigenome, for example, by the injection of mature transcriptomes into immature spermatozoa, can rescue normal development [18]. It is worth pointing out that, due to the exponential increase in complexity during embryonic development, the sooner an element perform its role, the more serious the result would be if it is altered [10]. For instance, the lack of miR-34c in spermatozoa causes imperfect first divisions of the embryo, hampering the rest of the process [28]. 

#### 3.2.6. Spermatozoa Epigenetics in Evolution

##### Environmental Factors

A phenotype arises by the combination of genotypic information and their expression induction by environmental stimuli [25]. Those inducers can proceed from the external environment, independent of the organism, or from genetic conditions of the organism that, together with the external environment, influence the corporal environment [1]. The corporal environment is compartmentalized into extracellular environments [9]. These local environments modulate cell epigenetics, determining its expression patterns. In this way, epigenetic-driven interaction between the environment and the genotype will define a phenotype [17,25,30]. SCs, Leydig cells, epididymal epithelia, and the female reproductive tract cells—from now on, the regulatory cells—are in charge of regulating the main environments to which sperm are exposed. For that reason, these are the cells that promote the establishment of epigenetic marks [10,17,24,51]. They can do so by employing RNAs, proteins, or signals that induce their synthesis and by the modification or the redistribution of other epigenetic elements [9,10,17,30]. Extracellular environment perturbations, by external or genetic factors, would influence those regulatory cells, possibly modifying spermatozoa epigenetic patterns [51]. The severity of the alterations would depend on the incidence phase, its intensity, and the path taken [1], and in some cases, it could generate reproductive problems [27]. It must be noted that epigenetics flexibility may allow partial revert to those situations by removing the environmental factors or compensating them [31,37]. 

Some of the factors that repeatedly have been bound to those disturbances are chemical contamination (fungicides, herbicides, pesticides, heavy metals, biopolymers, etc.) [5,17,25,27,51]; unhealthy eating habits (fat-rich diets or alcohol) and stress [10,16,18,24,25,27,30,32,33,36]; medical conditions and aging [28,32,45,52,53]. Their effects are several, including different types of infertility and pathologies in the descendants as cancer, syndromes or metabolic, cardiac, or autoimmune diseases, etc. [5,10,16,17,18,24,25,27,30,32,33,36,51]. A particular factor is the ARTs, especially IVF (in vitro fecundation) and ICSI (intracytoplasmic spermatozoa injection). There is evidence that its use influences spermatozoa epigenome, and individuals born using these means seem more susceptible to suffering certain illnesses [8,28,29,30,36,37]. However, there is no consensus about those findings because the interpretation of results shows controversy [43]. Additionally, there is an actual increase in the prevalence of some congenital pathologies due to these techniques. The gametes selection criteria do not really reflect the cell aptitude for development. Thus, enabling the formation of a zygote from germ cells may lack the normal epigenetic patterns, leading to the inheritance of epigenetic abnormalities [8,28,29,37]. 

##### Epigenetic Inheritance

The marks that overcome the reprogramming barriers will be inherited by descendants [10,16,31,33]. Theoretically, it can happen indefinitely if the mark keeps avoiding erasing. This has been observed for many generations, for example, in some transposon methylation marks [33]. That implies that the phenotype of an organism could be influenced by the environment that its ascendents were exposed to [10,27,33]. In this way, successive generations would possess a common phenotypic character that cannot be exclusively explained by genetic inheritance [16,23]; that is, epigenetic inheritance [10,27,31]. Epigenetic inheritance can be intergenerational (between two consecutive generations) or transgenerational (between no consecutive generations) [28,30]. Although the dynamic character of the epigenome [45] makes it probable that epigenetic inheritance has a lower relevance than embryo epigenetic acquirements, its influence has also been demonstrated [6,9,10,27,31,37]. It is still not known how epigenetic mechanisms would transmit a phenotype without loss of meaning between generations [10], but it seems that inheritance would be modulated by collaboration between cis and trans molecules [17,31]: the methylome and packaging proteins stability enables them to conserve information on a long-term basis [18,31]; while transcriptome and proteome, which undergo a less drastic transformation during genesis, may store epigenetic information in this period instead. Besides, their capability to move long distances also allows them to transfer environmental information to spermatozoa from other tissues [31]. When the modifications in the gamete affect essential and conserved aspects of development, epigenetic pathologies arise in the descendants, such as obesity, stress response, or neurological disorders [23,25,31]. If not, these regulatory changes could be neutral or even beneficial for the next generation and may provide them with adaptative advantages to the pre-existing environment [9]. 

##### Accelerated Evolution

The environmental effect in epigenetics [33] and its reversibility [29] may present adaptative intragenerational implications [4,10,30]. Taking also into account its heritability through germ cells, it arises the idea of “accelerated evolution”, whose attributes recall the evolution models of Landmark and the Pangenesis of Darwin and gives them a molecular explanation [10,21,27,50,54]. This phenomenon would consist of the organism’s adaptation to the environment by means of epigenetics and the subsequent transmission to the next generations via gametes of some of the acquired features (damaging, neutral or beneficial) [10,30,50]. Those adaptative responses will also suffer natural selection in the long term, changing their frequency in the population [4,50].

**Figure 7:**
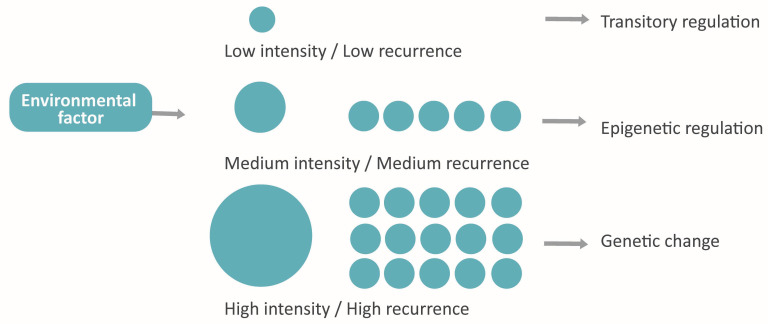
The hypothetical model proposed: Adaptative response conditioned by intensity and assiduity.

Moreover, the bidirectional influence between genome and epigenome [4,25,33,50] makes possible a new paradigm that connects the assiduity and the intensity of environmental factors with the nature of the adaptative response in following generations. Hypothetically, the factors with low frequency and intensity would induce a reversible epigenetic response, while the persistent or very strong factors would involve the genome and cause a stable adaptation (Figure 7).

## 4. Discussion

The results of this article show that spermatozoa epigenetics in humans is a recurring topic in scientific literature, and it is currently growing. This is congruent with assisted reproduction normalization as well as with epigenetics transversal expansion through other areas. Moreover, there are many countries and international collaborations involved in this study realm, and their publications usually are undertaken by high-impact editorials and journals. All of that reflects the importance of this study field for the scientific community. It is remarkable the presence of the methylome across the studies over the other mechanisms, a situation which may derive from its early description. The proteome, instead, is scarcely attended as a whole, probably due to its predominantly transversal character to the other mechanisms. Nowadays, publications are mainly focused on the bases of the subject, while applied research is hardly widespread. 

The interest in this study realm resides in the particularities of the gamete epigenome. Its establishment during the cell genesis is essential for the ulterior performance of the sperm role. For this reason, diseases and the external environment, which modify the spermatozoa extracellular environment, can have consequences on the individual fertility or its descendants’ phenotype. The lack of a detailed characterization of spermatozoa’s epigenetics and its influence pre and post-fecundation limits the accuracy level in assisted reproduction decision-making and treatments. This drives repeated reproductive failures and idiopathic cases. Epigenetics possesses the capacity to explain at a molecular level the phenotypic consequences of environmental factors. This fact opens the door to a series of applications in diagnostic and prognostic, prevention, and treatment at the spermatozoa level, as well as assisted reproduction techniques improvement. Currently, the cases of infertility or subfertility are addressed mainly by prevention strategies and by ART. These preventive measures range from general recommendations such as setting healthy dietary habits [17]; to guidelines focused on risk groups (professions with repeated exposure to damaging chemicals [5], patients with certain pathologies, or under some treatments [32]. The continuous nature of spermatogenesis during the reproductive life of the individual makes it viable and enough to employ the mentioned strategies in many cases. It is convenient making a commitment to the responsible management of patients with reproductive difficulties in order to constrain the use of ART to the indispensable cases.

In the future, research may allow the use of ART with minimal risk by reducing the exposure of the gamete to artificial environments, as well as increasing the robustness of the selection criteria by including additional perspectives such as epigenetic quality [8,29]. To do so, it would be necessary the development of standardized epigenetic markers that predict reproductive failure. Some of the candidates for this purpose are 6 mA (6-methyladenosine) in mRNA [6], 5 mC (5-methylcytosine) and 5 hmC (5-hydroxymethylcytosine) in DNA [51], H3K27 (methylation in Lys 27 of histone 3) [51], methylation of imprinted genes (H19, MEST, and SNRPN) [38], or the presence of certain ncRNA such as miRNA or circRNA (circular RNAs) [17,38]. These markers could also be useful in diagnostic and prognostic applications of reproductive problems and congenital pathologies with an epigenetic origin.

Additionally, studies in animal models have demonstrated that epigenetic activity can be oriented in the organism by means of supplementation [17,27], which may open the door to nonspecific treatments in the future. Patients whose pathologies are severe and irreversible would require the management of spermatozoa with epigenetic dysfunctions. Currently, as far as we know, these procedures have not been designed, but the flexibility of epigenetic mechanisms and the interaction of spermatozoa with the medium makes it possible the induction of nonspecific epigenetic changes by locating the gamete under a controlled environment. Moreover, the consolidation of genetic engineering techniques may allow specific epigenetic modifications, either at the spermatozoa or embryo level. Furthermore, the fast advance of technologies such as CRISPR or the development of Bioinformatics, Machine learning, and Big data [21,33,36] make it more suitable to focus on personalized medicine in this field [36].

An additional consideration is that epigenetic responses to the environment, after being sifted by natural selection, would have become an intragenerational and heritable adaptation, which would have implications for evolution. The total comprehension of this question requires explaining the information transference between environment, epigenetics, and phenotype, as well as between an individual and its descendants [10,31]. From this phenome arises the idea of a level of conservation of cell memory depending on the repetition or the strength of environmental stimuli. For this idea to be consistent, it would be needed to demonstrate by which means the cellular response acquires stability parallelly to the duration or potency of the external factor. 

Through this work, an overview of this study field has been provided, and a series of pending issues have been pointed out, such as establishing an agreed definition of “Epigenetics” and delimitating the mechanisms that compose it, examining the overlooked mechanisms deeply; applicating of epigenetic criteria in clinics, or studying how information is translated between environment and epigenetic mechanisms or between the last ones. These and other unsolved issues are an invitation to open new research lines and to continue filling in the gaps of knowledge.

## Data Availability

Not applicable.

## Supplementary Materials

The following supporting information can be downloaded at: (https://www.mdpi.com/article/10.3390/life13020364/s1), Figure S1: Summary of the activities and interdependence of epigenetic mechanisms; Figure S2: Proportion of documents of each journal and each publisher. There only appear the six journals and the four publishers with more publications; Table S1: Fundamental aspects to know about each epigenetic mechanism; Table S2: Number of publications obtained with each keyword and database after applying the exclusion criteria; Table S3: Number of publications obtained with each combination of keywords and database after applying the exclusion criteria; Table S4: Number of articles obtained with combinations of keywords after applying each criteria individually or simultaneously and number of wrongly accepted articles; Table S5: Number of publications in which each author appeared as a first author or not, and average of number of citations by paper and author. There only appear the six scientists with greater number of publications as a first author.
