# Supplementary material for: Epigenetics Role in Spermatozoa Function: Implications in Health and Evolution—An Overview
Source: Life (Basel). 2023 Jan 29;13(2):364. doi: 10.3390/life13020364 (PMC9964922; doi:10.3390/life13020364)
Supplement: Supplementary file 1 [file life-13-00364-s001.zip › life-2144108-supplementary.pdf]

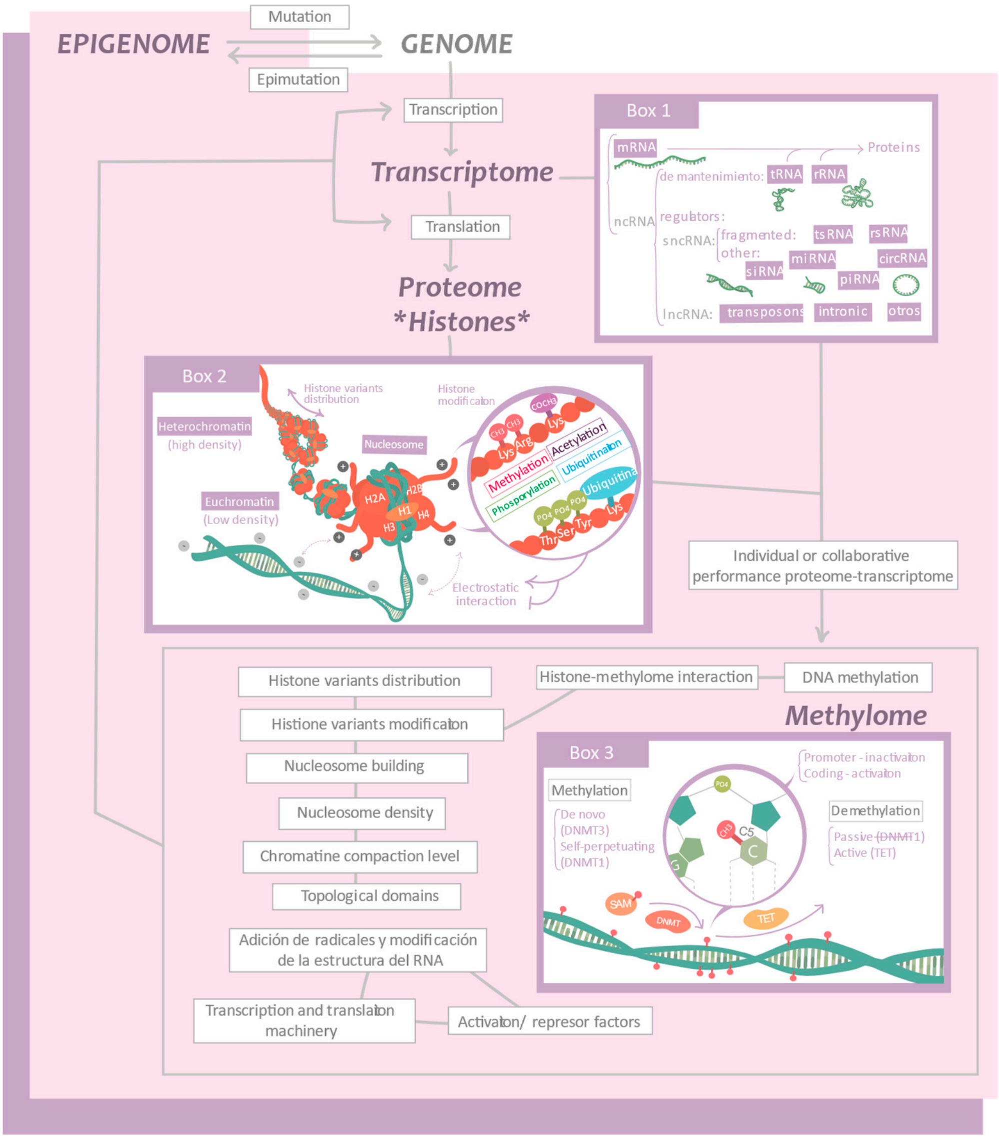

Figure S1. Summary of the activities and interdependence of epigenetic mechanisms.

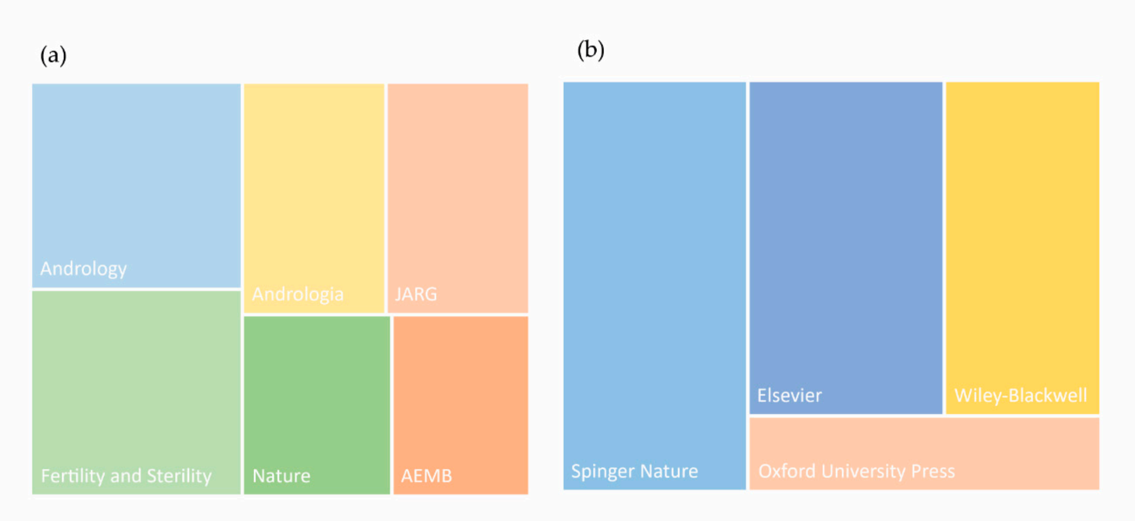

Figure S2. The proportion of documents of each journal (**a**) and each publisher (**b**) (JARG=Journal of Assisted Reproduction and Genetics; AEMB= Advances in Experimental Medicine and Biology). There only appear the six journals and the four publishers with more publications.

Table S1. Fundamental aspects about epigenetic mechanisms.

Epigenetic Mechanisms	Basic Dynamics	Main Functions	Primary Effectors	Particularities
DNA methylation	Covalent addition of a methyl group from SAM* to a Cys in CpG islands.	To vary the accessibility of expression machinery to cis elements in the sequence.	DNMT* and TET*	Imprinted regions have different methylation between the male and female gametes.
Packaging proteins	Formation of nucleosomes by interaction** with DNA.	To vary the accessibility of expression machinery to wide regions in the genome.	Histones***	The most common histone modifications are acetylation, methylation, phosphorylation, and ubiquitination
Transcriptome	Interaction by sequence complementarity or by 3D conformation properties.	To vary the availability of mRNA or to modify chromatin structure.	ncRNA****	Each RNA subtype has a specific function.
Proteome	Modulate other epigenetic mechanisms. *****	Intermediate between mechanisms.	Proteins	-

* SAM = S-adenosyl methionine; DNMT =methyltransferase (methylation); TET = translocation enzyme (demethylation). ** Electrostatic interaction thanks to the basicity of packaging proteins. *** Histones are the most common but not the only packaging proteins; histone density differentiates heterochromatin and euchromatin; histone variants appear in different tissues; histone chemic modifications change their basicity level. **** ncRNA = non-coding RNA; ncRNA are divided into maintenance RNA (tRNA and rRNA) and regulatory RNA, subdivided into long and short ncRNA (lncRNA and sncRNA); RNA chemic modifications change their properties. ***** Ex.: Formation of complexes with ncRNA, distribution of histones, modification of histones or RNA,etc.

Table S2. The number of publications obtained with each keyword and database after applying the exclusion criteria.

Keywords	Pubmed	Scopus	WOS
Assisted reproductive techniques	631	1168	869
Epigenetics	5543	54212	1849
Male infertility	3994	10852	5974
Paternal inheritance	237	469	275
Spermatozoa	3793	13812	9526

WOS = Web of Science.

Table S3. The number of publications obtained with each combination of keywords and database after applying the exclusion criteria.

Keywords	PubMed	Scopus	WOS
Epigenetics + Male infertility	56	293	106
Epigenetics + Paternal inheritance	44	71	21
Epigenetics + Spermatozoa	51	491	222
Epigenetics + Spermatozoa + Assisted reproductive techniques	0	106	883

WOS = Web of Science.

Table S4. The number of articles obtained with combinations of keywords after applying each criteria individually or simultaneously, and the number of wrongly accepted articles.

Criteria Applied	Accepted	Wrongly Accepted
2011-2022 only	4351	0
Human only	3490	58
English only	5466	4
All	2344	-

Table S5. The number of publications in which each author appeared as a first author or not, and the average number of citations by paper and author. There only appear the six scientists with greater number of publications as a first author.

Authors	Articles as a First Author	All Articles	Citation Average
Carrell, D.T.	4	31	48
Denomme, M.	4	4	35
Gunes, S.	4	6	33
Hotaling, J.M.	4	16	32
Jenkins, T.G.G.	12	22	45
Wu, H.	4	6	25
